# Accuracy of Bruch’s membrane opening minimum rim width and retinal nerve fiber layer thickness in glaucoma diagnosis depending on optic disc size

**DOI:** 10.1007/s00417-024-06375-3

**Published:** 2024-01-19

**Authors:** Verena Anna Englmaier, Jens Julian Storp, Martin Dominik Leclaire, Larissa Lahme, Viktoria Constanze Brücher, Julia Biermann, Raphael Diener, Nicole Eter

**Affiliations:** Department of Ophthalmology, University Medical Center Munster, Albert-Schweitzer-Campus 1, Building D15, 48149 Munster, Germany

**Keywords:** Glaucoma, Diagnosis, Optic coherence tomography, RNFL, BMO-MRW, Microdiscs, Macrodiscs

## Abstract

**Background/aim:**

The aim of this paper is to compare retinal nerve fiber layer thickness (RNFL) and Bruch’s membrane opening-based minimum rim width (BMO-MRW) in terms of their performance in detecting early and moderate/advanced glaucoma using receiver operating characteristics (ROC) analysis and the classification using the 5th percentile as a cut-off.

**Methods:**

One hundred eyes from 100 patients with early glaucoma (mean deviation (MD): < -5.0 dB) and 100 eyes from 100 patients with moderate/advanced glaucoma (MD: > -5.0 dB) were carefully matched to healthy controls based on optic disc size. Then, the dataset was divided, based on the 50th percentile of the measured Bruch’s membrane opening area (BMO-A), into small (BMO-A < 1.95 mm^2^) and large optic discs (BMO-A > 1.95 mm^2^). Finally, the discriminative performance of BMO-MRW and RNFL between glaucoma and controls using ROC analysis and the manufacturer’s classification based on the 5th percentile was analyzed.

**Results:**

In discriminating between glaucoma and matched healthy controls, global BMO-MRW and global RNFL thickness had comparable areas under the ROC curve for eyes with early glaucoma and both small BMO-As (ROC ± confidence interval [CI] 0.91 [0.87 to 0.95] and 0.88 [0.83 to 0.93]) and large BMO-As (0.86 [0.82 to 0.92] and 0.84 [0.79 to 0.90]), as well as in moderate/advanced glaucoma with small BMO-As (0.99 [0.98 to 1.00] and 0.97 [0.95 to 1.00]) and large BMO-As (0.94 [0.91 to 0.98] and 0.97 [0.94 to 1.00]). Using the calculated 5th percentile as a threshold value, the sensitivities for the detection of early and moderate/advanced glaucoma were comparable for BMO-MRW and RNFL in eyes with small optic discs (early glaucoma: fifty-two percent and 61%; moderate/advanced glaucoma: ninety-one percent and 92%). In eyes with large optic discs, the sensitivity of BMO-MRW was inferior to that of RNFL for both early (38% versus 51%) and moderate/advanced (80% versus 91%) glaucoma.

**Conclusion:**

Based on an ROC analysis, the discriminative performance of BMO-MRW and RNFL between patients with early and moderate/advanced glaucoma and a healthy control group matched based on optic disc size is comparable in eyes with BMO-As smaller and larger 1.95 mm^2^. Using a classification based on the 5th percentile, as used in clinical practice, RNFL is shown to be superior to BMO-MRW regarding sensitivity in glaucoma detection with large optic discs. This study underscores the importance of RNFL imaging and measurement in the diagnostic evaluation of glaucoma, especially in cases of large optic discs.


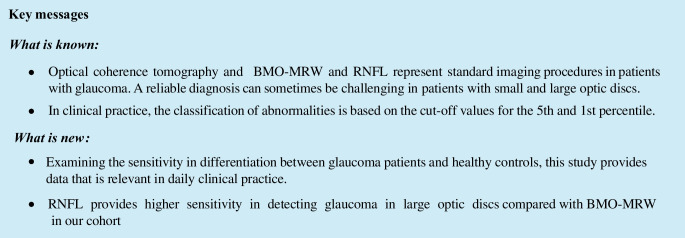


## Introduction

Open-angle glaucoma is a progressive optic neuropathy affecting millions worldwide and characterized by retinal ganglion cell loss [1, 2], which manifests as structural changes in the optic nerve head (ONH), with retinal nerve fiber layer (RNFL) thickness loss and neuroretinal rim thinning, causing functional deficits [3]. Optical coherence tomography (OCT) of the ONH is an objective method that allows the early identification of this structural damage so that serious visual impairment can be prevented via the early initiation of treatment [4, 5].

Bruch’s membrane opening-based minimum rim width (BMO-MRW) and peripapillary RNFL are frequently used in clinical practice [6], and the understanding of the diagnostic performance of these parameters in the detection of glaucoma is largely derived from ROC analysis [7]. Here, BMO-MRW is mentioned as a parameter with superior [6] or equivalent [8–10] diagnostic capability as compared to the RNFL thickness (RNFLT) in discriminating between glaucoma cases and healthy controls.

However, in clinical practice, the classification of ONH abnormalities is analyzed using diagnostic classification reports, which are based on the 5th and 1st percentiles. Zheng et al. found that, based on the manufacturer’s classification report, RNFL had a higher sensitivity in diagnosing glaucoma than BMO-MRW [7], which stands in contrast with the ROC-analysis results in the literature.

One caveat in this regard is that BMO-MRW can vary as a function of BMO size: eyes with smaller disc sizes will be expected to have thicker MRW measurements as compared with eyes with larger disc sizes given the same number of ganglion cells. Therefore, the normative data on one-dimensional BMO-MRW vary strongly with disc size and differ significantly between small and large discs [11], which is why the performance of BMO-MRW and RNFLT with micro- and macrodiscs has, thus far, been analyzed independently. In eyes with small optic discs, or microdiscs, similar performance levels on the part of BMO-MRW and RNFL in glaucoma detection have been described [12, 13], whereas the data on their performance in eyes with large optic discs, or macrodiscs, are contradictory [12, 14]. The question that arises is that of whether the ROC analysis reflects the actual performance in everyday clinical practice in eyes with small and large optic discs. Thus, the aim of this study was to analyze the performance of RNFL and BMO-MRW in glaucoma detection using both ROC analysis and classification based on percentiles depending on optic disc size.

## Methods

### Design and setting

This retrospective, monocentric study was approved by the ethics committee of the Medical Association of Westfalen-Lippe and the University of Münster and adhered to the tenants of the Declaration of Helsinki.

### Primary endpoint

The primary endpoint of this study is to compare global peripapillary RNFLT and global BMO-MRW regarding their performance in glaucoma detection in eyes with small and large optic disc sizes using (1) ROC analysis and (2) a classification report based on the manufacturer’s 5th percentile.

#### Dataset

##### Patient selection

The dataset used included 200 eyes from 200 patients with open-angle glaucoma (the glaucoma group) and 200 eyes from 200 healthy patients (the control group) who underwent consultation at the University Eye Hospital Münster between January 2018 and December 2022.

The glaucoma group consisted of patients with open-angle glaucoma; a history of intraocular pressure (IOP) above 24 mmHg; the need for anti-glaucomatous therapy; disease onset in adulthood; an open, inconspicuous chamber angle; and the absence of other causes of so-called secondary open-angle glaucoma. The diagnosis was confirmed in clinical practice by a glaucoma expert from our department. The control group consisted of healthy controls with no suspicious findings regarding glaucoma or other ocular diseases. By definition, none of the controls were receiving treatment for glaucoma, were being treated for ocular hypertension, or had a history of IOP above 21 mmHg.

##### Patient examinations

All patients received a comprehensive clinical examination, which included a detailed medical history, the refraction of the eye, best corrected visual acuity measurement, slit-lamp biomicroscopy, Goldmann applanation tonometry, and the indirect ophthalmoscopy of the optic nerve head. All eyes had structural measures of the ONH as obtained with spectral domain optical coherence tomography (Spectralis®, Heidelberg Engineering GmbH, Heidelberg, Germany). Visual field analysis was performed with an automated Humphrey Visual Field Analyzer II (HFA II, model 750; Carl Zeiss Meditec AG, Jena, Germany) using the standard program of the 30–2 Swedish interactive threshold algorithm (SITA fast).

##### Matching of the glaucoma and control groups

The 200 eyes from 200 glaucoma patients were matched to a healthy control group of 200 eyes from 200 patients based on their Bruch’s membrane opening area (BMO-A). The matching accuracy for every eye was ± 0.05 mm^2^. Thus, the dataset consisted of 200 eyes from glaucoma patients with various BMO-As and 200 eyes from healthy patients with accurately (± 0.05mm^2^) matched BMO-As.

##### Subdivision of the dataset

**Fig. 1:**
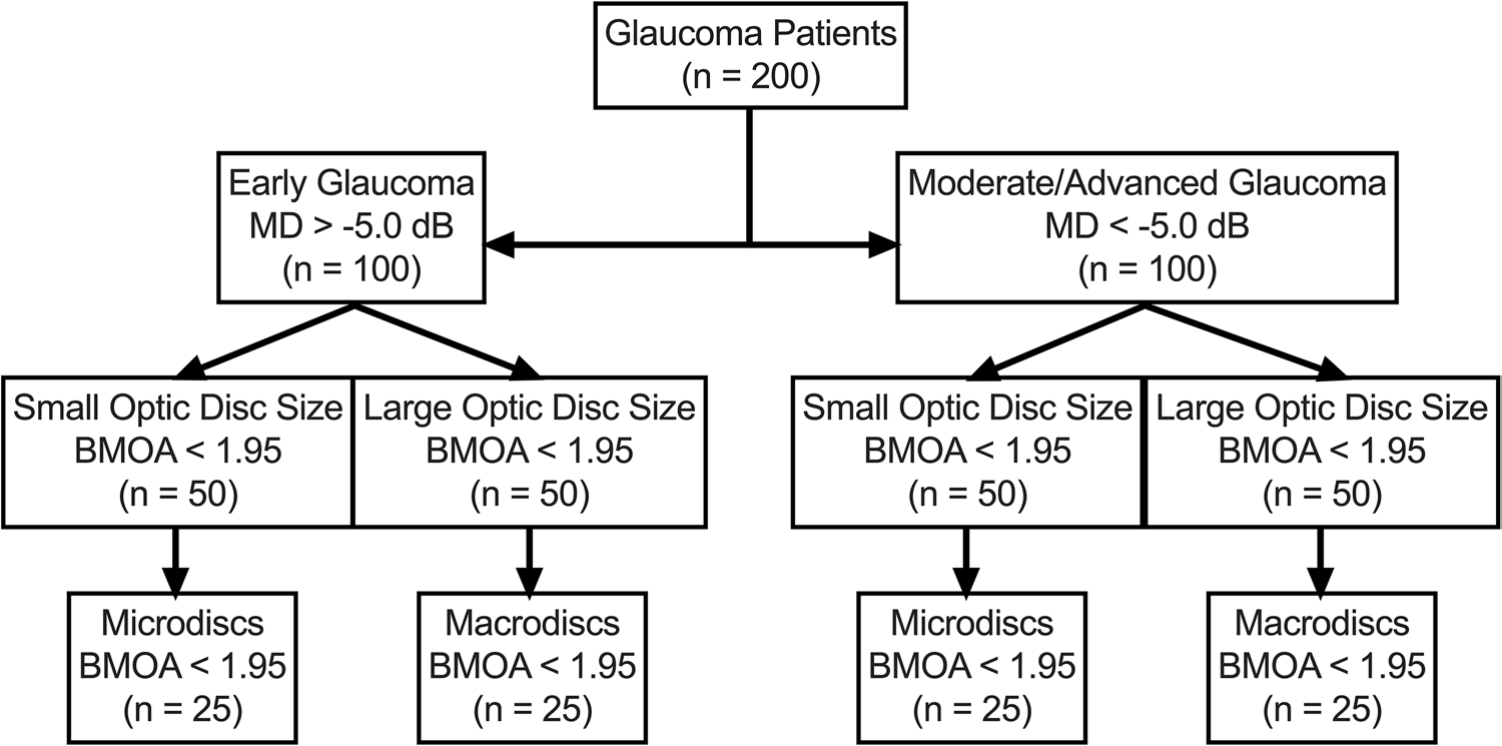
Flowchart of the subdivision of the patients in their subgroups according to disease stadium (early glaucoma or moderate/advanced glaucoma, according to the MD) and the optic disc size according to BMO-A

The dataset was then divided into patients with early (MD > -5.0 dB) and moderate/advanced glaucoma (MD < -5.0 dB) according to the mean deviation (MD) of the perimetry similar to the publication of Millis et al. [15]. Based on BMO-A, these two groups were then again divided into two different groups, those with (1) small and (2) large optic discs, based on the 50% percentile and will be referred to as that in the following. Finally, the twenty-five smallest optic discs of the group of eyes with small optic discs (microdiscs) and twenty-five largest eyes of the group of eyes with large optic discs (macrodiscs) were analysed in a subgroup analysis. The subdivision flow can be seen in Fig. 1.

## Analysis

Data were recorded in Microsoft Office Excel (Microsoft, Redmond, WA, USA) (2023). Statistical analysis was performed using SPSS (IBM SPSS Statistics 28). The graphic representation was implemented with GraphPad Prism 9 (GraphPad Software, Inc., California, USA). The level of statistical significance was set at P ≤ 0.05. Inferential statistics are intended to be exploratory, not confirmatory, and were interpreted accordingly.

The normality of the data distribution was tested using the Kolmogorov–Smirnov test, and the Mann–Whitney U Test was used to compare baseline data. Continuous variables were tested for mean differences using ANOVA. Dichotomous variables were tested with Pearson X^2^ Test. Fisher’s exact test was used to calculate the sensitivities and specificities for the classification report.

### ROC analysis

The diagnostic accuracy of each parameter was evaluated by constructing receiver operating characteristic (ROC) curves by plotting the true-positive rate (sensitivity) against the false-positive rate (1-specificity). The calculated area under the ROC curve (AUC) shows the diagnostic accuracy of each parameter, with 1.0 representing excellent accuracy and 0.5 indicating no discriminative characteristics.

#### BMO-a-matched control group

In the first ROC analysis, the performance of measured global BMO-MRW and global RNFL in discriminating glaucoma patients from healthy BMO-A-matched controls was investigated.

#### BMO-a-matched and age-adjusted control group

In a second ROC analysis, the control group’s measured RNFL and BMO-MRW were adjusted based on the age of the matched glaucoma patients. The recalculated values take into account an RNFL decrease of 0.23 µm and a BMO-MRW decrease of 1.77 µm per year [16]. The difference in the patient’s age was multiplied by the mentioned factors, and the calculated values were subtracted from the measured RNFLT or BMO-MRW for control patients who were younger then and added for control patients who were older than the matched glaucoma patients. The age-adjusted RNFL and BMO-MRW values are referred to as aRNFL and aBMO-MRW.

### Classification based on percentiles

The limits calculated by the manufacturer for the 5th percentile depend on both the age of the patient, as well as on the size of the optic disc. With increasing age, the BMO-MRW and RNFLT threshold values decrease. With increasing disc size, the BMO-MRW cut-off values decrease, whereas the RNFL increases. We approximated the manufacturer’s limits using a multiple linear regression. The formula used in the calculation of thresholds is listed in Table 1.

**Table 1:** Formula for the calculation of 5th-percentile threshold values

5% Percentile Threshold	Multiple Linear Regression Formula
Y = RNFL	Y = 87.54 + 3.628*BMO-A + -0.189*Age
Y = BMO-MRW	Y = 407.6 + -48.77*BMO-A + -1.337*Age

*BMO-MRW* Bruch’s membrane opening based-minimal rim width, *RNFL* retinal nerve fiber layer thickness, *BMO-A* Bruch´s membrane opening area.

Subsequently, the sensitivities and specificities based on the calculated threshold values for the 5th percentile were calculated for each included patient. The measured RNFL and BMO-MRW values were compared to the calculated threshold values and evaluated as being below (diseased) or above (healthy) the 5th percentile.

## Results

### Patient characteristics

The participants’ demographic and clinical characteristics at OCT measurement are shown in Table 2. There were no significant differences in laterality and spherical refraction between the groups, whereas a statistically significant difference in age was found.

**Table 2:** Demographic and clinical characteristics of the subjects

	Early Glaucoma	Matched Control	Moderate/Advanced Glaucoma	Matched Control	*P*
Patients	100	100	100	100	> 0.05
Eyes	100	100	100	100	> 0.05
Laterality, R/L	47/53	52/48	51/49	44/56	> 0.05
Age, y	67 ± 14	45 ± 20	70 ± 12	45 ± 19	** < 0.001**
Refraction, D					
Spherical	-0.61 ± 2.13	-0.06 ± 2.51	-0.02 ± 2.52	-0.10 ± 2.06	> 0.05
Cylindrical	-0.17 ± 0.99	-0.84 ± 0.88	-1.17 ± 0.98	-0.87 ± 0.82	** < 0.001**
Visual Field, MD	-0.84 ± 1.37	-	-11.50 ± 7.32	-	** < 0.001**

Data are means ± SD values, except laterality, patients, and number of eyes. Continuous variables were tested for mean differences with ANOVA. Dichotomous variables were tested with Pearson X^2^ Test. Visual field results are depicted as mean deviation.

The morphometric characteristics of the patients with early and moderate/advanced glaucoma with micro- and macrodiscs are not shown.

### Morphometric characteristics

The BMO-MRW decreased with increasing optic disc size in early glaucoma patients (BMO-A < 1.95 mm^2^: 250 ± 54 µm, BMO-A > 1.95 mm^2^ 223 ± 58 µm), as well as in the matched control groups for both early glaucoma (BMO-A < 1.95 mm^2^: 339 ± 74 µm, BMO-A > 1.95 mm^2^: 229 ± 75 µm) and moderate/advanced glaucoma (BMO-A < 1.95 mm^2^: 360 ± 77 µm, BMO-A > 1.95 mm^2^: 269 ± 83 µm). In eyes with moderate/advanced glaucoma, MRW did not differ between eyes with moderate/advanced glaucoma and small (BMO < 1.95 mm^2^: 171 ± 55 µm) or large optic discs (BMOA > 1.95 mm^2^: 178 ± 98 µm).

The RNFLT increased with increasing optic disc size in all groups, including early (BMO-A < 1.95 mm^2^: 79 ± 11 µm, BMO-A > 1.95 mm^2^: 85 ± 14 µm) and moderate/advanced glaucoma (BMO-A < 1.95 mm^2^: 62 ± 15 µm, BMO-A > 1.95 mm^2^: 63 ± 22 µm), as well as in the matched control groups for early glaucoma (BMO-A < 1.95 mm^2^: 94 ± 10 µm, BMO-A > 1.95 mm^2^: 98 ± 12 µm) and moderate/advanced glaucoma (BMO-A < 1.95 mm^2^: 95 ± 9 µm, BMO-A > 1.95 mm^2^: 101 ± 8 µm).

The morphometric characteristics are summarized in Tables 3 and 4.

**Table 3:** Morphometric characteristics of the patients with early glaucoma and the matched control group

	Early Glaucoma		Matched Control		*P*
	Small BMO-A < 1.95 mm^2^	Large BMO-A > 1.95 mm^2^	Small BMO-A < 1.95 mm^2^	Large BMO-A > 1.95 mm^2^	
*N*	50	50	50	50	na
BMO-A, mm^2^	1.64 ± 0.20	2.35 ± 0.28	1.64 ± 0.19	2.35 ± 0.27	na
BMO-MRW, µm	250 ± 54	223 ± 58	339 ± 74	292 ± 75	** < 0.001**
aBMO-MRW, µm	-	-	305 ± 21	251 ± 89	** < 0.001**
RNFL, µm	79 ± 11	85 ± 14	94 ± 10	98 ± 12	** < 0.001**
aRNFL, µm	-	-	89 ± 11	93 ± 12	** < 0.001**

Data are means ± SD values. Continuous variables were tested for differences in means with ANOVA. *na* not analyzed, *n* number, *aBMO-MRW* age adjusted BMO-MRW.

**Table 4:** Morphometric characteristics of the patients with moderate/advanced glaucoma and the matched control group

	Moderate/Advanced Glaucoma		Matched Control		*P*
	Small BMO-A < 1.95 mm^2^	Large BMO-A > 1.95 mm^2^	Small BMO-A < 1.95 mm^2^	Large BMO-A > 1.95 mm^2^	
*n*	50	50	50	50	na
BMO-A, mm^2^	1.68 ± 0.19	2.30 ± 0.28	1.68 ± 0.18	2.30 ± 0.28	na
BMO-MRW, µm	171 ± 55	178 ± 98	360 ± 77	314 ± 61	** < 0.001**
aBMO-MRW, µm	-	-	318 ± 76	269 ± 83	** < 0.001**
RNFL, µm	62 ± 15	63 ± 22	95 ± 9	101 ± 8	** < 0.001**
aRNFL, µm	-	-	90 ± 9	95 ± 10	** < 0.001**

Data are means ± SD values. Continuous variables were tested for mean differences with ANOVA. *na* not analyzed, *n* number, *aBMO-MRW* age-adjusted BMO-MRW, *aRNFLT* age-adjusted RNFL thickness.

The morphometric characteristics of the patients with early and moderate/advanced glaucoma with micro- and macrodiscs are not shown.

### ROC analysis

#### BMO-a-matched control group

In discriminating between cases of glaucoma and matched healthy controls, global BMO-MRW and global RNFL thickness present comparable AUROCs in eyes with early glaucoma and both small BMO-As (AUROC ± confidence interval [CI] 0.83 [0.75 to 0.90] and 0.84 [0.76 to 0.92]) and large BMO-As (0.77 [0.68 to 0.86] and 0.77 [0.68 to 0.86]).

In distinguishing eyes with moderate/advanced glaucoma from matched healthy controls, global BMO-MRW and RNFL showed higher AUROCs as compared to early glaucoma. The results were comparable for RNFL and BMO-MRW in eyes with small BMO-As (0.98 [0.97 to 0.99] and 0.95 [0.92 to 0.99]) and large BMO-As (0.91 [0.85 to 0.98] and 0.95 [0.90 to 1.00]).

#### BMO-a-matched and age-adjusted control group

The performance of BMO-MRW and RNFL in discriminating between eyes with early and moderate/advanced glaucoma and the age-matched control groups was, for both RNFL and BMO-MRW, lower as compared to the performance with the non-age-matched control group. In eyes with a small optic disc size (BMO-A < 1.95 mm^2^), BMO-MRW and RNFL showed comparable AUROCs in eyes with early (0.70 [0.59 to 0.80] and 0.75 [0.65 to 0.84]) and moderate/advanced glaucoma (0.95 [0.91 to 0.99]) and 0.92 [0.87 to 0.98]).

**Fig. 2:**
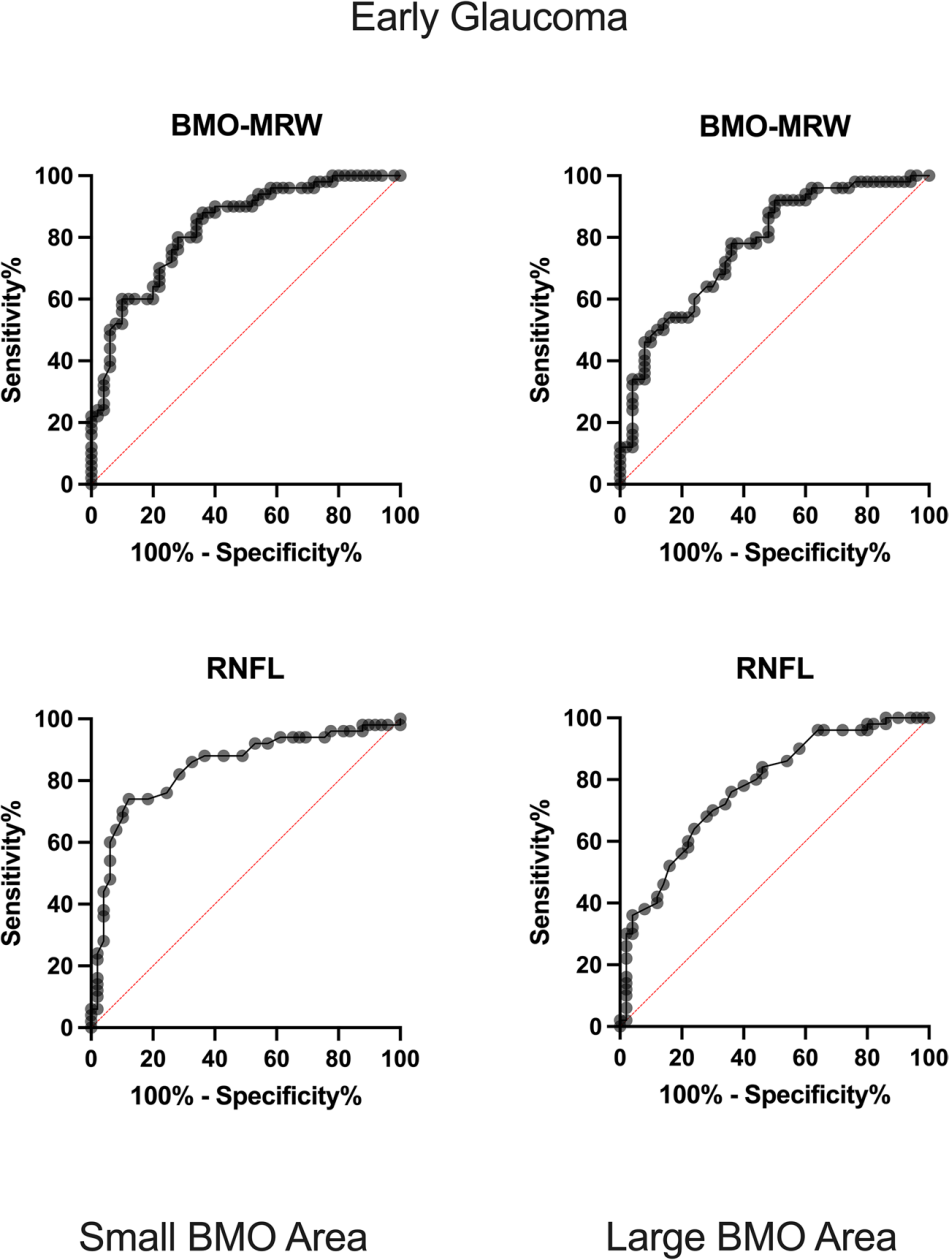
ROC curves for the detection of early glaucoma. On the left side, ROC curves for small BMO-A can be seen. Top left: BMO-MRW for small BMO-A and bottom left: RNFL for small BMO-A. On the right side, ROC curves for large BMO-A are displayed. Top right: BMO-MRW for large BMO-A. Bottom right: RNFL for large BMO-A. X-axis shows the specificity as percentages from 0 to 100%; y-axis shows the sensitivity recorded as percentages from 0 to 100%. BMO-MRW: Bruch’s membrane opening-based minimum rim width; RNFL: retinal nerve fiber layer; BMO: Bruch’s membrane opening

**Fig. 3:**
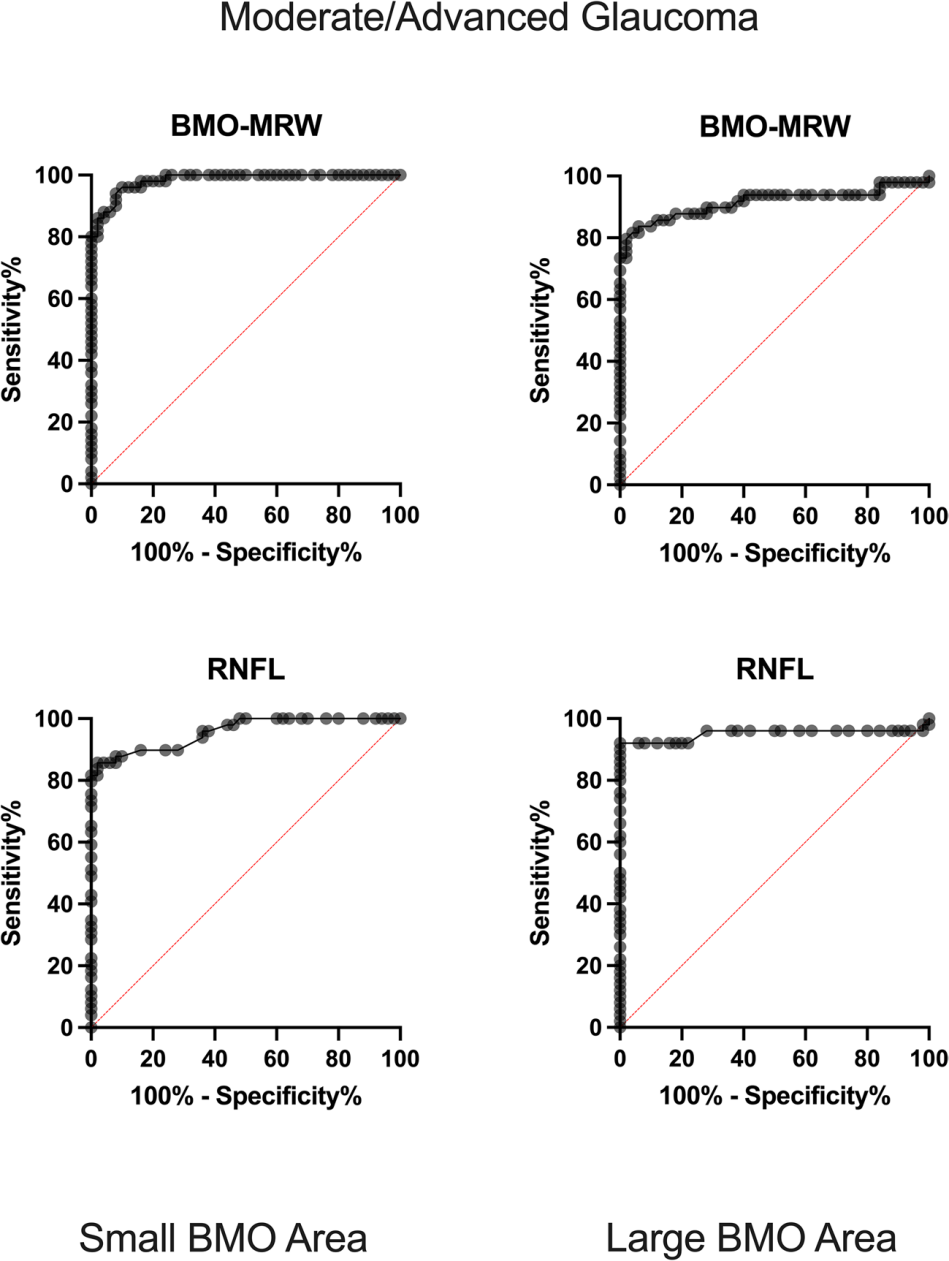
ROC curves for the detection for moderate/advanced glaucoma. On the left side, curves for small BMO-A can be seen. Top left: BMO-MRW for small BMO-A. Bottom left: RNFL for small BMO-A. On the right side, ROC curves for large BMO-A are displayed. Top right: BMO-MRW for large BMO-A. Bottom right: RNFL for large BMO-A. X-axis shows the specificity recorded as percentages from 0 to 100%; y-axis shows sensitivity recorded as percentages from 0 to 100%. BMO-MRW: Bruch’s membrane opening-based minimum rim width; RNFL: retinal nerve fiber layer; BMO: Bruch’s membrane opening

In eyes with large optic discs (BMO-A > 1.95 mm^2^), the AUROCs for BMO-MRW were inferior to those of RNFL in both early glaucoma (0.59 [0.47 to 0.70] and 0.65 [0.55 to 0.77]) and moderate/advanced glaucoma (0.82 [0.73 to 0.90]) and 0.92 [0.86 to 0.99]). Figures 2 and 3 show the ROC-Curves.

The results are summarized in Table 5.

**Table 5:** Diagnostic performance of BMO-MRW and RNFL in detecting early and moderate/advanced glaucoma based on an ROC analysis

	ROC Analysis			
	Early Glaucoma		Moderate/Advanced Glaucoma	
	Small BMO-A < 1.95 mm^2^	Large BMO-A > 1.95 mm^2^	Small BMO-A < 1.95 mm^2^	Large BMO-A > 1.95 mm^2^
*n*	50	50	50	50
AUROC BMO-MRW	0.83 (0.75 to 0.90)	0.77 (0.68 to 0.86)	0.98 (0.97 to 1.00)	0.91 (0.85 to 0.98)
AUROC RNFLT	0.84 (0.76 to 0.92)	0.77 (0.68 to 0.86)	0.95 (0.92 to 0.99)	0.95 (0.90 to 1.00)
BMO-MRW Sensitivity	38%	34%	88%	82%
RNFL Sensitivity	44%	36%	85%	92%
AUROC aBMO-MRW	0.70 (0.59 to 0.80)	0.59 (0.47 to 0.70)	0.95 (0.91 to 0.99)	0.82 (0.73 to 0.90)
AUROC aRNFL	0.75 (0.65 to 0.84)	0.65 (0.55 to 0.77)	0.92 (0.87 to 0.98)	0.92 (0.86 to 0.99)
aBMO-MRW Sensitivity	6%	4%	72%	51%
aRNFL Sensitivity	24%	16%	75%	82%

Area-under-the-receiver-operating-characteristic curve values and 95% CIs, as well as sensitivity at a fixed specificity of 95%, for patients with early glaucoma and moderate/advanced glaucoma. *aBMO-MRW* age-adjusted BMO-MRW, *aRNFLT* age-adjusted RNFL thickness.

### Classification analysis

Using the calculated 5th percentile as a threshold value, the sensitivities for the detection of early and moderate/advanced glaucoma were comparable for BMO-MRW and RNFL in eyes with small optic discs (early glaucoma: 52% and 56%, respectively; moderate/advanced glaucoma: 86% and 86%, respectively). In eyes with large optic discs, the sensitivity of BMO-MRW was inferior to that of RNFL in both early (42% versus 46%) and moderate/advanced (68% versus 84%) glaucoma. The results are summarized in Table 6.

**Table 6:** Diagnostic performance of BMO-MRW and RNFL in detecting early and moderate/advanced glaucoma using a classification analysis based on the threshold value of the 5th percentile

	Classification Analysis Based on 5th Percentile			
	Early Glaucoma		Moderate/Advanced Glaucoma	
	Small BMO-A < 1.95 mm^2^	Large BMO-A > 1.95 mm^2^	Small BMO-A < 1.95 mm^2^	Large BMO-A > 1.95 mm^2^
*n*	50	50	50	50
	Sensitivity			
BMO-MRW	52%	42%	86%	68%
RNFL	56%	46%	86%	84%
	Specificity			
BMO-MRW	80%	72%	94%	86%
RNFL	78%	78%	100%	98%

*BMO-MRW* Bruch’s membrane opening based-minimal rim width, *RNFL* retinal nerve fibre layer thickness, *BMO-A* Bruch’s membrane opening area.

## Subgroup analysis of micro- and macrodiscs

### ROC analysis

#### BMO-a matched control group

In discriminating between cases of glaucoma and matched healthy controls, global BMO-MRW and global RNFL thickness present comparable AUROCs in eyes with early glaucoma and both microdiscs (AUROC ± confidence interval [CI] 0.87 [0.77 to 0.96] and 0.88 [0.78 to 0.98]) and macrodiscs (0.71 [0.58 to 0.86] and 0.74 [0.61 to 0.88]).

In distinguishing eyes with moderate/advanced glaucoma from matched healthy controls, global BMO-MRW and RNFL showed higher AUROCs as compared to early glaucoma. The results were comparable for RNFL and BMO-MRW in eyes with microdiscs (0.99 [0.96 to 1.00] and 0.99 [0.99 to 1.00]) and macrodiscs (0.88 [0.77 to 1.00] and 0.92 [0.81 to 1.00]; (See Table 7).

**Table 7:** Diagnostic performance of BMO-MRW and RNFL in detecting early and moderate/advanced glaucoma in micro- and macrodiscs based on an ROC analysis

	ROC Analysis			
	Early Glaucoma		Moderate/Advanced Glaucoma	
	Microdiscs < 1.69 mm^2^	Macrodiscs > 2,35 mm^2^	Microdiscs < 1.69 mm^2^	Macrodiscs > 2,35 mm^2^
*n*	25	25	25	25
AUROC BMO-MRW	0.87 (0.77 to 0.96)	0.71 (0.58 to 0.86)	0.99 (0.96 to 1.0)	0.88 (0.77 to 1.00)
AUROC RNFLT	0.88 (0.78 to 0.98)	0.74 (0.61 to 0.88)	0.99 (0.99 to 1.00)	0.92 (0.81 to 1.00)
BMO-MRW Sensitivity	60%	28%	92%	75%
RNFL Sensitivity	44%	24%	96%	92%

Area-under-the-receiver-operating-characteristic curve values and 95% CIs, as well as sensitivity at a fixed specificity for 95%, for patients with early glaucoma and moderate/advanced glaucoma.

#### BMO-a matched and age adjusted control group

Data not shown.

### Classification analysis

Using the calculated 5th percentile as a threshold value with the exception of microdiscs in eyes with moderate/advanced glaucoma, the sensitivities of RNFL in glaucoma detection was shown to be superior to that of BMO-MRW in micro- and macrodiscs in early and moderate/advanced glaucoma. The results are summarized in Table 8.

**Table 8:** Diagnostic performance of BMO-MRW and RNFL in detecting early and moderate/advanced glaucoma in micro- and macrodiscs using a classification analysis based on the threshold value of the 5th percentile

	Classification Analysis Based on 5th Percentile			
	Early Glaucoma		Moderate/Advanced Glaucoma	
	Microdiscs < 1.69 mm^2^	Macrodiscs > 2.35 mm^2^	Microdiscs < 1.69 mm^2^	Macrodiscs > 2.35 mm^2^
*n*	25	25	25	25
	Sensitivity			
BMO-MRW	54%	32%	94%	78%
RNFL	72%	56%	95%	90%
	Specificity			
BMO-MRW	100%	100%	100%	98%
RNFL	92%	100%	96%	96%

*BMO-MRW* Bruch’s membrane opening based-minimal rim width, *RNFL* retinal nerve fibre layer thickness, *BMO-A* Bruch’s membrane opening area.

## Discussion

While the performance levels of BMO-MRW and RNFL in discriminating between early and moderate/advanced glaucoma and healthy disc-size-matched patients were comparable based on an ROC analysis of eyes with small (BMO-A < 1.95 mm^2^) and large optic discs (BMO-A > 1.95 mm^2^), the classification report using the 5th percentile as a cut-off RNFL value showed higher sensitivity in eyes with a large optic disc size as compared to that using BMO-MRW. Because an assessment performed by means of percentiles, as opposed to an ROC analysis, is used in clinical practice, the results of the study are highly relevant to everyday clinical practice. In the following, the aforementioned results are discussed.

In eyes with a small BMO-As (< 1.95 mm^2^), we found that BMO-MRW and RNFL thickness had comparable AUCs for discriminating between 50 eyes with early (0.83 and 0.84) glaucoma and 50 eyes with moderate/advanced glaucoma (0.98 and 0.95) and a healthy optic-disc-size-matched control group. This result was also confirmed in a subgroup analysis of eyes with a disc size < 1.69 mm^2^. These results are in line with the literature:

For microdiscs, Enders et al. found that RNFL and BMO-MRW had similar performance in discriminating 51 glaucomatous patients from 20 healthy controls with small optic discs, with AUCs of 0.81 and 0.87, respectively. Also, similar to our results, they reported comparable sensitivities (RNFL = 66.4%; BMO-MRW = 68.6%) at a fixed specificity of 95% [13]. However, optic disc size was determined via HRT (< 1.63 mm^2^), making a comparison with the present study difficult.

Similar to our work, Gmeiner et al. analyzed eyes with a small BMO-A (< 1.84 mm^2^) and found a comparable AUCs for BMO-MRW and RNFLT in discriminating between 23 eyes with pre-perimetric (0.904 and 0.857, respectively) and 23 eyes with perimetric glaucoma (1.00 and 0.987, respectively). The sensitivities at a fixed specificity of 95% were even higher for BMO-MRW as compared to RNFLT in both pre-perimetric (69.6% versus 47.8%) and perimetric glaucoma (100% versus 87.0%) [12]. Thus, based on the ROC analysis, there is comparable discriminative performance between glaucoma patients and healthy controls using BMO-MRW and RNFL.

In eyes with a large BMO-A (> 1.95 mm^2^), in our work, ROC curves demonstrated that RNFL tended to perform similar or slightly better than BMO-MRW. The subgroup analysis of optic discs with a BMO-A above 2.35 mm^2^ showed a similar result.

In the literature, contradictory results can be found regarding the performance of RNFL and BMO in detecting glaucoma in eyes with large optic discs. Similar to our work, Gmeiner et al. stated that the discriminative performance levels of BMO-MRW and RNFL in eyes with a BMO-Area > 1.84 mm^2^ were comparable in 27 eyes with pre-perimetric (0.764 versus 0.817, respectively) and 19 eyes with perimetric glaucoma (0.892 versus 0.934, respectively). The sensitivities at a fixed specificity were even higher for RNFL in both pre-perimetric (49.7% versus 25.9%) and perimetric glaucoma (78.9% versus 57.9%) [12], which can also be confirmed in our work, as seen in Table 5.

In contrast to that, Enders et al. concluded that the performance of BMO-MRW in discriminating 44 eyes with glaucoma from 70 healthy controls was higher (AUC = 0.96) than that of RNFL (AUC = 0.89) in eyes with BMO-A > 2.45 mm^2^ [17].

These contradictory results may be explained by the different age distributions of the study populations. The age of the control group used by Enders et al. was lower (38.2 ± 25 years) than the age of the glaucoma group (65.0 ± 11.0 years) [14]. In contrast, Gmeiner et al. found no difference between healthy controls and patients with glaucoma [12]. Because the age-related decrease in MRW is higher (1.77 µm/year) than that of RNFL (0.23 µm/year), this may have led to easier discrimination between controls and glaucoma patients in Enders et al.. Similarly, in our work, after adjusting the parameter based on age, RNFL showed better performance than MRW.

In clinical practice, the classification of healthy and diseased participants is based on using the manufacturer’s 5th percentile as a threshold. We found that, in eyes with small optic discs, RNFL and BMO-MRW had comparable results, whereas in eyes with large optic discs, RNFL showed higher sensitivity for both early glaucoma (46% versus 42%) and moderate/advanced glaucoma (84% versus 68%, respectively). This effect could also be shown amplified in the subgroup analysis of micro- and macrodiscs. This stands in contrast to the results of the ROC analysis in the above mentioned literature. Zheng et al. analyzed the performance of RNFL and BMO-MRW independent of disc size and also found that using the 5th percentile to classify 188 eyes from 137 patients with glaucoma showed a higher sensitivity of 88.83% for RNFL as compared to a sensitivity of 76.06% for BMO-MRW [7]. To the best of our knowledge no other comparable studies can be found in the literature.

However, an exact comparison between ROC analysis and classification based on percentiles is difficult because they are fundamentally different. The performance of the ROC analysis depends on the age and optic disc size distribution in the control group and the severity of the disease in the glaucoma group. In contrast, both age and optic disc size are included in the calculation of the 5th percentile. This has the advantage being more independent from the study population analyzed.

The poorer performance of BMO-MRW with large or very large ONHs may be due to various factors. In large optic discs, the BMO-MRW is thinner overall, as the axons entering the optic nerve head are distributed over a wider rim area [13, 14]. Therefore, large but healthy discs may be erroneously classified as glaucomatous. This, however, leads to a decrease in specificity, not in sensitivity, and only explains why many patients with large optic discs are classified as diseased in clinical practice [18], even though they are healthy, whereas a decrease in sensitivity is not explained.

As the sensitivity of BMO-MRW is lower with large optic discs, apparently, BMO-MRW falls below the 5th percentile later in the course of glaucoma than RNFL; thus, more patients with glaucoma are classified as healthy when using BMO-MRW as compared to RNFL. This may be explained by the reactivity of the tissue to the progression of glaucoma. Amini et al. hypothesized that the remodeling of neural tissues could occur more slowly in large optic discs as glaucoma advances [19]. Similar, Vianna et al. found that higher baseline values of BMO-MRW, because they were present in small optic discs, were related to faster reductions [20]. Thus, in large optic discs, slower reduction would be expected, which could lead to a subsequent drop below the 5th percentile. Furthermore, larger discs could contain a higher proportion of non-neuronal tissue, as the axons are distributed over a wider rim area [19]. A decrease in neural tissue could be distorted by a larger proportion of non-neuronal tissue, leading to falsely high values. Similar, Mardin et al. found that the accuracy of BMO-MRW could be worsened by retinal blood vessels, in contrast to that of RNFL measurements [21]. This could, however, explain why RNFL shows a higher sensitivity than BMO-MRW in diagnosing glaucoma in eyes with large optic discs.

Interestingly, when analyzing microdiscs in early glaucoma, sensitivities of RNFL were superior (60%) to that of BMO-MRW (44%). We hypothesize that there is more neuronal tissue in small optic nerves and that the disease has not yet progressed to the point of falling below the 5th percentile.

Our work has several limitations. First, due to the characteristics of the study population and the use of CSLT or BMO area for the calculation of optic disc size, it is difficult to compare our work with studies in the literature. Ideally, there would be an even distribution of glaucoma, as well as an age- and optic-disc-size-matched control group. Therefore, in our study, a BMO-A matched control group was used, which is a strength of our study. However, there was a significant age difference between the healthy and glaucoma populations, which clearly influenced the results of our work. In a second ROC analysis, we adjusted the parameters based on age-related changes [16]. Because the adjustment of age is only a theoretical calculation and the effect of age on the parameter may also differ between small and large optic nerves, this limited the results to a certain extent. However, no contradictory results were found after adjusting the parameters based on age.

Second, in using the 5th percentile to analyze the performance of the parameters to detect glaucoma, a mode of analysis was chosen that was more independent of the study population, compared to ROC analysis. This is an advantage of the study. However, the cut-off values for the 5th percentile were calculated approximately, by means of a regression. Because the manufacturer’s exact formula is not known, this is a limitation of the study, as it could have influenced the results.

Third, our classification of the severity of glaucoma is based on the mean deviation as proposed by Mills et al. [15]. However, we only classified into early (MD: < -5.0 dB) and moderate or advanced glaucoma (MD: > -5.0 dB). The results of the study in advanced glaucoma should therefore be interpreted with caution.

Fourth, both the intraocular pressure and axial length were not analysed in our study using OCT data. As this significantly influences the RNFL and BMO-MRW measurements [22], this might have influenced the results to a certain extent. In particular, the influence of high myopia or hyperopia on the accuracy of RNFL and BMO-MRW classification based on the 5th percentile needs further investigation. The results of this study should be applied with caution to highly myopic and highly hyperopic eyes.

Fifth, one major limitation is the retrospective design. Further prospective studies are needed. Finally, our work addresses the topic of disc dependency in glaucoma detection using BMO-MRW and RNFL, which has been addressed using BMO-MRA in eyes with small and large optic disc sizes [11]. However, this parameter is not widely used in everyday clinical practice. A promising approach is the use of ganglion cell layer thickness [18], but threshold values have not yet been established for both BMO-MRA and ganglion cell layer thickness.

In conclusion, based on an ROC analysis, the discriminative performance levels of BMO-MRW and RNFL between patients with early and moderate/advanced glaucoma and a healthy control group matched based on optic disc size were comparable in eyes with BMO-As smaller and larger 1.95 mm^2^. Using a classification based on the 5th percentile, as is used in clinical practice, RNFL was shown to be superior to BMO-MRW in terms of sensitivity in glaucoma diagnosis with large optic discs. Our findings underscore the importance of RNFL imaging and measurement in the diagnostic evaluation of glaucoma, especially in eyes with large optic discs and a BMO-A above 2.35 mm^2^.

## Data Availability

The datasets which were analyzed during the current study are available from the corresponding author on reasonable request.

## Abbreviations

*RNFL*: Retinal nerve fiber layer
*RNFLT*: Retinal nerve fiber layer thickness
*BMO*: Bruch’s membrane opening
*BMO-MRW*: Bruch’s membrane opening minimum rim width
*BMO-A*: Bruch’s membrane opening area
*ROC*: Receiver operating characteristics
*AUC*: Area under the curve
*AUROC*: Area under the receiver operating characteristics
*CSLT*: Confocal scanning laser tomography
*na*: Not applicable
*aBMO-MRW*: Age-adjusted BMO-MRW
*aRNFLT*: Age-adjusted RNFL thickness
